# Soil organic matter and clay zeta potential influence aggregation of a clayey red soil (Ultisol) under long-term fertilization

**DOI:** 10.1038/s41598-021-99769-w

**Published:** 2021-10-15

**Authors:** Yangbo He, Mingxuan Yang, Rui Huang, Yao Wang, Waqar Ali

**Affiliations:** Key Laboratory of Arable Land Conservation, Ministry of Agriculture and Rural Affairs, Huazhong Agricultural University, Wuhan, Hubei China

**Keywords:** Agroecology, Geochemistry

## Abstract

The effect of soil organic matter (SOM) on aggregation of variably-charged red soils (Ultisol) through clay zeta potential is not fully understood. Therefore, the objectives of this study were to investigate the SOM effect on the clay zeta potential and soil aggregation after fertilization. Soils under 17 years of fertilization (manure, NPK + straw, NPK, and control (CK) were adjusted by KCl solution to reach varying soil pH and concentration in order to determine clay zeta potential, cations, and aggregate size distribution. The SOM content and C-functional groups by ^13^C-NMR analysis were also determined. Results showed that the negative zeta potential displayed a bell-shaped pattern with increasing concentration of KCl, but displayed different amplitude of variation among treatments. Manure had the highest zeta potential value and its degree of variation in relative to the value at KCl concentration of 0.1 mol L^−1^ (19%), NPK + straw and NPK treatments were similar, and CK was the least. Greater negative zeta potential for manure treatment was attributed to higher SOM content, aromatic-C functional groups, and their greater concentrations of Ca^2+^ and Mg^2+^ than did the CK. As a result, higher SOM and clay zeta potential yielded in less release of amount of soil particles (< 10 μm) (*r* = − 0.46*) and enhanced water stable macroaggregates for manure instead of NPK + straw. Long-term manure fertilization would be suggested as a conservation practice for red soil due to its increase in soil aggregate stability and negative zeta potential in subtropical climate.

## Introduction

Stable aggregates are important to improve soil permeability and restrict soil erosion. Aggregates of red soil (Ultisol), as an important source for crop production in large areas of subtropical climate of China, were dominated by high proportion of microaggregates^1^. High proportions of microaggregates of red soil made these soils prone to erosion in rain season^2^, and prone to drought stress in seasonal drought periods due to the decreased ability to store plant available water^3,4^. Therefore, proper management of red soil and understanding the mechanism of soil aggregate development/breakdown isnecessary to facilitate soil physical–chemical processes.

Application of amendments including manure and crop straws have been proven to be effective agricultural practices when attempting to improve soil aggregation and crop yields^1^. Pig manure has improved macroaggregate percentage and aggregate associated C and N content^5,6^. Soybean green manure improved large aggregates (> 2 mm) greater than a control treatment^5^. Also, rice straw incorporation alone or in compound with NPK fertilizers was generally effective in increasing water-stable aggregation^1^, and restricting release of small particles (< 10 µm) of an Inceptisols, due to an increase in soil organic carbon (SOC)^7^. In contrast, Yu et al.^8^ determined that one year after incorporation of rice straw in an Ultisol displayed greater microaggregate (< 5 µm) release than the control. Similarly, Claremont clayey soil having high SOC (2.2%) in Australia also showed higher clay dispersion than Urrbrae sandy loam soil (SOC = 1.4%) at the pH of 11^9^. Hence, amendment-initiated changes in soil aggregates is complex and multifaceted in various climate and might also be determined by variations in soil organic matter and soil minerals.

Variations of soil aggregation after amendments can be addressed to differences in soil cementing materials (i.e. soil organic matter (SOM), soil oxides, and SOM-oxides complex), the subsequent differences in soil surface charge properties and internal forces. For example, free and occluded light SOC played major roles in macroaggregate stability of a Mollisol^10,11^, probably because the positive effect of SOC on van der Waals attractive force between soil particles can limit the release of microaggregate^7^. However, improvement in SOC cannot always result in decreasing in microaggreate portion or re-flocculation of small soil particles. For example, an Ultisol having high SOC after straw treatments enhanced the release of small particles (< 5 µm)^8^. Small particles of some types of Ferralsols with high SOC in subtropical climate of Brazil were difficult to re-flocculate when they were released from mechanical breakdown of macroaggreates^12^. There must exist other soil properties that limit the roles of SOC and induce repulsive force prevailing attractive force between soil particles. Ultiol or Oxisols in these studies possessed high amount of variably-charged soil oxides compared to soils in temperate regions having 2:1 type of clay minerals with permanent charges^13^, which might change the intensity of imbalance in charge on soil particles and the intenal forces between particles**.** Therefore, it is necessary to understand the variably-charged soil minerals’ interaction with SOM and their subsequent effect on soil surface electrochemical properties such as zeta potential (a main factor influencing soil internal force).

Due to the interaction between SOM and oxides, soil surface charge number and surface charge density will change and thus influence the zeta potential. The variable charges of SOM and Fe/Al oxides can also result in a stronger overlapping of oppositely charged electric double layers than between 1:1 clay minerals and oxides in subtropical soils^14^. As an example, the electrostatic repulsive force between adjacent soil particles can be weakened less severely in the presence of a high content of SOM in subtropical soils than temperate soils^8^. Also, negative zeta potential of two Ultisols and an Oxisol variably increased in response to increase in solution ionic strength because ionic strength attenuated the imbalance of negative surface charges' effect on zeta potential^15^. A decrease in negative zeta potential was generally consistent with an increase in clay dispersion, confirming the important zeta potential effect on soil structure^9^.

In subtropical climates, soil solution concentration after fertilization (i.e. N) easily varied with high wetting and drying cycles in field^16–18^. Variation of soil ionic strength may counterbalance the negative charges from SOM, and then influence the extent of imbalance in charges and zeta potential. However, the content and types of SOM that result from long-term fertilization of a red soil has not yet been well documented, and the subsequent effect on zeta potential and soil structure is not clear. We hypothesize that organic fertilization that increase mostly in SOM will decrease the negative zeta potential of red soil and then increase the amount of microaggregates. Therefore, the objectives of this study were: (1) to investigate the SOM effect on zeta potential after long-term fertilization (manure, NPK + straw, NPK and CK), and (2) to identify SOM and zeta potential effect on soil aggregation.

## Results

### Soil organic matter properties

**Figure 1:**
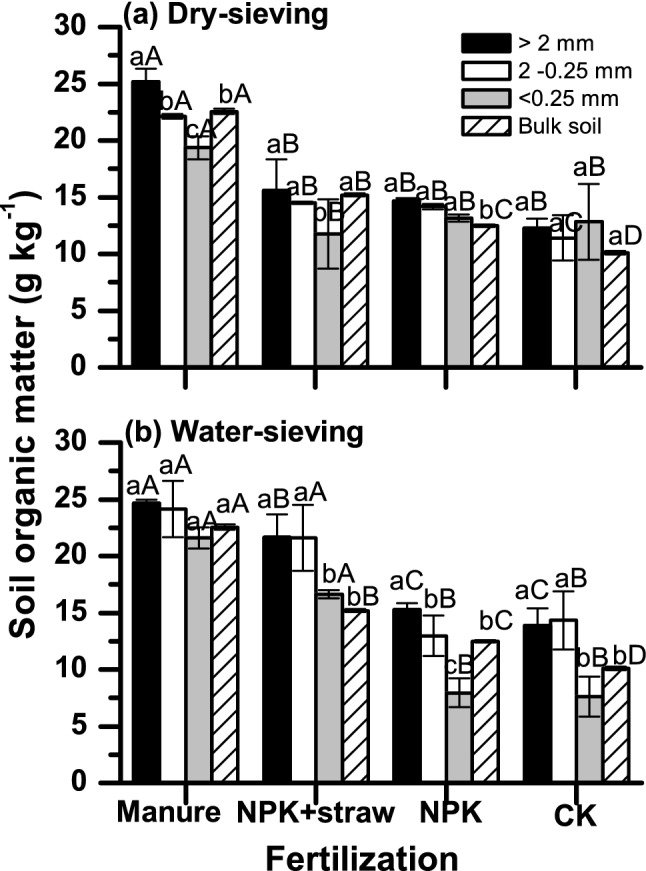
Soil organic matter (SOM) distribution on different aggregate size across treatments at (**a**) dry sieving and (**b**) water sieving. Different lower-case letters indicate significant differences of SOM among aggregate size under the same of fertilization. Different capitalized letters indicate significant differences of SOM among the four types of fertilization at the same aggregate size.

Fertilization significantly affected the SOM content in bulk soils and in aggregates (Fig. 1a,b). Concentrations of SOM in bulk soil were manure > NPK + straw > NPK > CK. Compared to CK, SOM increased by 125%, 54.6% and 0.3% for manure, NPK + straw and NPK, respectively. In addition, after water-sieving, the SOM content was consistently higher in macroaggregates (> 2 mm) than microaggregates (< 0.25 mm), irrespective of the fertilization type (Fig. 1b). For example, for CK, the SOM decreased almost 50% from macroaggregates to microaggregates. Compared to CK, the decline of SOM with aggregate size was less, with 7.6% and 39.1% decline for manure and NPK + straw, respectively. Similar SOM distribution patterns occurred among dry-sieved aggregate sizes.

**Figure 2:**
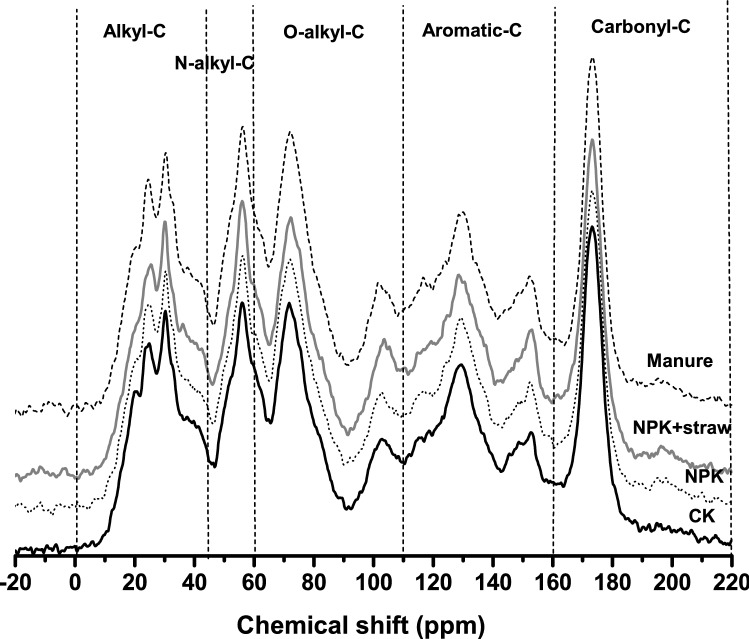
CP /MAS ^13^C-NMR spectrum characteristics of humic acid in red soil under four fertilization treatments.

The CP/MAS ^13^C-NMR spectra of the bulk soil under each treatment was shown in Fig. 2, and the relative percentage of OC functional groups were summarized in Table 1. Aromatic-C, O-alkyl-C, Alkyl-C and Carboxylic-C were the major OC functional groups. Among them, aromatic-C had the highest percentage (24.7% to 26%). Slightly higher aromatic-C and lower Carbonyl-C exhibited after NPK + straw than CK, but these two C functional groups were similar among manure, NPK and CK treatments (Table 1).

**Table 1:** Relative percentages of functional groups with different chemical shift intervals in the CPMAS13C-NMR spectra in humic acid of red soil under fertilization treatments.

Treatment	Alkyl C (%)	Methoxyl/N-alkyl C (%)	O-alkylC (%)	Aromatic C (%)	CarbonylC (%)	Aliphatic C/aromatic C	Alkyl C/O-Alkyl C	Hydrophobic -C/hydrophilic-C
Manure	20.77	10.92	26.20	24.57	17.54	1.89	0.82	0.84
NPK + straw	20.33	10.87	26.16	26.08	16.56	1.87	0.79	0.83
NPK	20.55	10.62	26.16	24.94	17.72	1.91	0.79	0.83
CK	21.01	10.67	25.62	24.69	18.00	1.78	0.78	0.87

Alkyl-C, Methoxyl/N-alkyl-C, O-alkyl-C, Aromatic-C and Carbonyl-C were identified with chemical shift of 0–45, 45–60, 60–110, 110–160, and 160–220 ppm, respectively.

Aliphatic C/Aromatic C = (Alkyl C + O-alkyl C)/Aromatic C;

Hydrophobic-C/Hydrophilic-C = (Alkyl C + Aromatic C)/(Methoxyl /N-alkyl C + O-alkyl C + Carbonyl C).

### Change of soil pH, cations and clay zeta potential under fertilization

**Figure 3:**
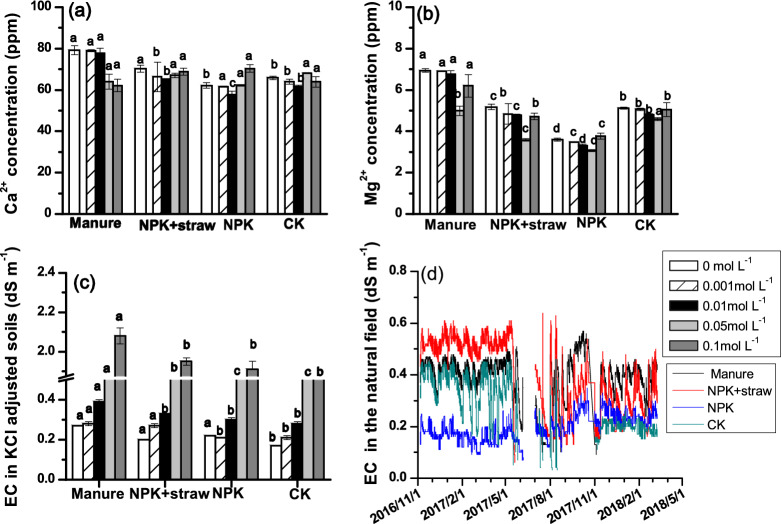
The solution information under four fertilization treatments (**a**) Ca^2+^ concentration, (**b**) Mg^2+^ concentration, (**c**) EC values in laboratory, (**d**) EC values in field. Different lower-case letters indicate significant differences of Ca^2+^, Mg^2+^ and EC in laboratory among fertilization type under the same KCl concentration.

Soil pH firstly increased followed by slight decrease with increased concentrations of KCl equilibration under each fertilization type, where the inflection point occurred at 0.01 mol L^−1^ (Table 2). CK had soil pH ranging from 6.2 to 6.6 when KCl concentration increased from 0 to 0.1 mol L^−1^. Compared to CK, pH generally followed an order of Manure > NPK + straw > NPK at the same KCl concentration. The magnitude variation of soil pH as concentration of KCl increased was also greater under fertilization treatments than CK. For example, when KCl concentration increased from 0 to 0.1 mol L^−1^, pH increased by 14.1%, 7.5%, 7.6% and 6.5% for manure, NPK + straw, NPK and CK, respectively (Table 2). The EC, Ca^2+^and Mg^2+^ concentration also displayed greater values in organic treatments than that in CK under the same KCl concentration (Fig. 3).

**Table 2:** Soil pH variation with KCl solution concentration under the fertilizer treatments.

Fertilization treatment	KCl concentration (mol L^−1^)				
	0	0.001	0.01	0.05	0.1
Manure	6.4b	6.7a	7.3a	6.9a	6.3a
NPK + straw	6.7a	6.6a	7.2a	6.9a	6.3a
NPK	6.6a	6.6a	7.6a	6.8a	6.0b
CK	6.2c	6.2b	6.6b	6.4b	6.0b

Different lowercase letters indicate significant differences of pH between the fertilization treatments across each KCl concentration.

**Figure 4:**
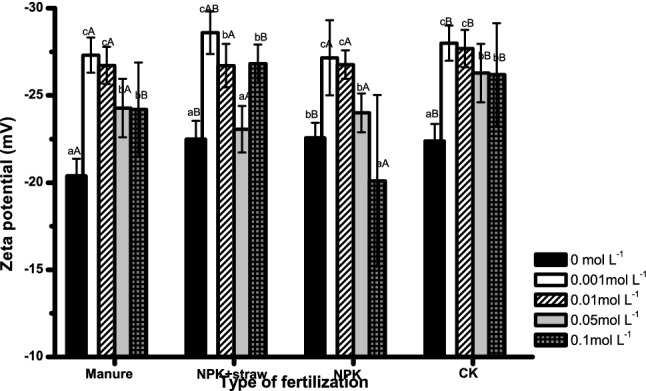
Clay zeta potential in response to KCl concentration over all fertilizations. Different lower-case letters indicate significant differences with concentration under each type of fertilization. Different capitalized letters indicate significant differences among the four types of fertilization at the same concentration.

Clay zeta potential changed in different patterns among fertilization treatments due to their differences in SOM content, pH and cations (Fig. 4). Firstly, clay negative zeta potential significantly decreased and then increased with the increase of KCl concentration under all treatments. The lowest negative zeta potential value occurred around 0.001 mol L^−1^, which was generally consistent with the inflection concentration point for pH (at 0.01 mol L^−1^), because zeta potential showed no significant differences at KCl concentration of 0.001 and 0.01 mol L^−1^ (Fig. 4). However, with the increase of KCl concentration, the change in zeta potential displayed greater magnitude in organic treatments (manure, NPK + straw) than others. For example, when concentration increased from 0 to 0.001, and from 0.001 to 0.05 mol L^−1^, the negative zeta potential decreased by 34% (from − 20.4 to − 27.3 mV), 27% (− 22.5 to − 28.6 mV), 20% (− 22.6 to − 27.2 mV) and 25% (from − 22.4 to − 28.0 mV), and then increased by 11%, 6%, 26% and 6%, for manure, NPK + straw, NPK and CK, respectively. Secondly, at the same KCl concentration, negative zeta potential value was generally significantly higher under manure than CK, while exhibited no significant differences among manure, NPK + straw and NPK (except for at 0 and 0.1 mol L^−1^).

### Soil aggregation and grain size distribution

**Figure 5:**
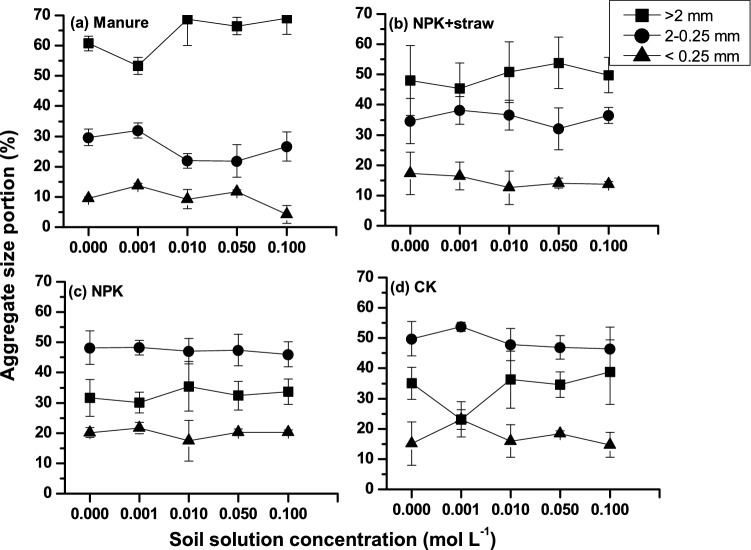
Aggregate size distribution with concentration over four types of fertilizations, including (**a**) Manure, (**b**) NPK + straw, (**c**) NPK, (**d**) CK.

The soil aggregate was dominated by size > 2 mm for manure and NPK + straw, while aggregates was dominated by size 2–0.25 mm for NPK and CK (Fig. 5). The > 2 mm macroaggregate portion displayed a slight decrease with the increase of concentration from 0 to 0.001 mol L^−1^, while microaggregate (< 0.25 mm) displayed an opposite pattern (Fig. 5). Also, the change of aggregate portion with concentration depended on fertilization types. For example, for aggregates (< 0.25 mm), manure and NPK + straw yielded in smaller percentage values and smaller degree of change with increased concentration compared to CK (Fig. 5a,b). When concentration increased from 0 to 0.001 mol L^−1^, < 0.25 mm portion increased from 9.6 to 13.7% (magnitude 43.3%), from 17.3 to 16.4% (-5%), and from 15.2 to 23.1% (52.4%) for manure, NPK + straw and CK, respectively.

**Figure 6:**
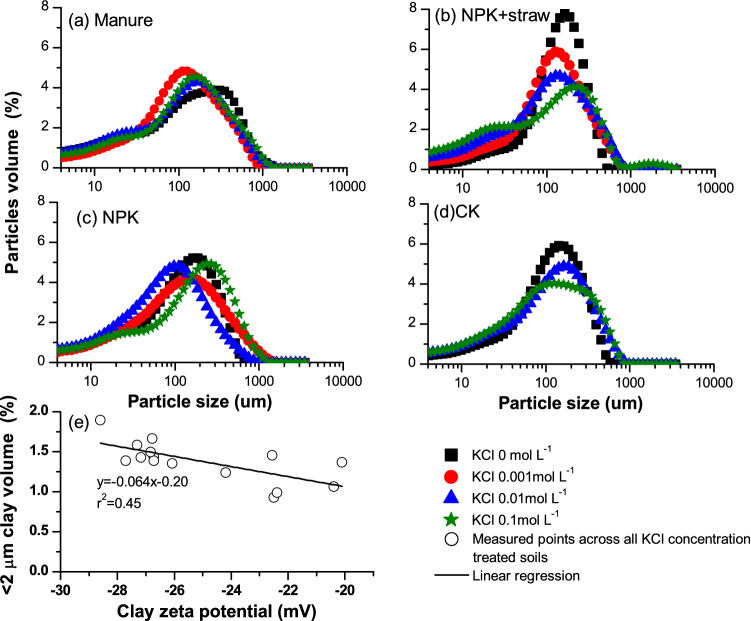
Grain size distributions with concentration by laser diffraction under different fertilizations, including (**a**) Manure, (**b**) NPK + straw, (**c**) NPK, (**d**) CK. Data for CK under 0.001 mol L^−1^ was lost. (**e**) Regression relationship between particles (< 2 μm) and clay zeta potential across all fertilization types.

The microaggregate portion change with concentration was further displayed through laser diffraction size analysis in Fig. 6. Similarly as the pattern of  < 0.25 mm microaggregate in response to the increase in concentration in Fig. 5, grains (< 100 μm) also increased with concentration from 0 to 0.001 mol L^−1^ due to the effect of zeta potential. When concentration increased from 0 to 0.001 mol L^−1^, grains (< 2 μm) changed from 1.07 to 1.29%, from 0.43 to 0.90%, from 1.46 to 1.43% for manure, NPK + straw and NPK, respectively. A significant regression further demonstrated the negative effect of zeta potential on clay dispersion (< 2 μm) (r^2^ = 0.45). Obvious difference in particles (20–40 μm) also existed across concentrations.

### Relationship between aggregate size and SOM, zeta potential

The SOM on each aggregate size was negatively correlated with grains (< 20 μm) percentage for manure and NPK treatment (Table 3). For example, the *r* between SOM on aggregate (< 0.25 mm) and grain percentage (< 5 and 10–20 μm) was − 0.46 and − 0.45 for manure treatment. Zeta potential was also significantly negatively correlated with grains < 100 μm. Especially, zeta potential obviously influenced grains (< 10 μm) for manure, and grains (20–40 μm) for NPK and CK. This indicated that a substantial decrease of SOM and zeta potential were conductive to release of soil microaggregate.

**Table 3:** Correlation among the measured soil parameters from all concentration under each type of fertilization.

Fertilizer type		> 2 mm	2–0.25 mm	< 0.25 mm	MWD (mm)	< 5 μm volume	5–10 μm volume	10–20 μm volume	20–40 μm volume	40–100 μm volume
Manure	Zeta potential	0.04	0.08	− 0.16	− 0.09	− 0.47*	− 0.46*	− 0.35	0.09	− 0.38
	pH	0.20	− 0.41	0.09	0.04	0.51*	0.56*	0.57*	0.78**	− 0.15
	SOM on > 2 mm	0.17	− 0.16	− 0.12	0.07	− 0.46*	0.26	− 0.45*	− 0.35	0.25
	SOM on 2–0.25 mm	− 0.16	0.16	0.12	− 0.08	− 0.46*	− 0.26	− 0.45*	− 0.35	− 0.25
	SOM on < 0.25 mm	− 0.15	0.15	0.10	− 0.09	− 0.45*	− 0.26	− 0.45*	− 0.35	− 0.25
NPK + straw	Zeta potential	− 0.27	0.24	0.26	− 0.21	− 0.15	0.18	0.18	0.15	− 0.25
	pH	0.49*	0.51*	0.35	0.46*	0.53**	0.57**	0.54**	0.50*	0.27
	SOM on > 2 mm	0.06	0.05	− 0.19	0.11	− 0.19	− 0.11	− 0.22	− 0.12	− 0.25
	SOM on 2–0.25 mm	0.08	0.03	− 0.21	0.14	− 0.19	− 0.11	− 0.22	− 0.12	− 0.25
	SOM on < 0.25 mm	0.04	0.07	− 0.17	− 0.06	− 0.19	− 0.11	− 0.22	− 0.12	− 0.24
NPK	Zeta potential	− 0.20	− 0.06	0.30	− 0.24	− 0.06	− 0.12	− 0.27	− 0.56**	− 0.45*
	pH	0.41*	− 0.09	0.51*	0.53**	0.21	0.34	0.53**	0.74**	0.68**
	SOM on > 2 mm	0.15	− 0.45	0.27	0.00	− 0.79**	− 0.68**	− 0.70**	− 0.31	− 0.19
	SOM on 2–0.25 mm	− 0.08	0.15	− 0.05	− 0.02	0.32	0.24	0.29	0.13	0.07
	SOM on < 0.25 mm	0.14	− 0.46	0.28	0.00	− 0.78**	− 0.68**	− 0.68**	− 0.30	− 0.19
CK	Zeta potential	0.03	− 0.04	− 0.01	0.08	− 0.64**	− 0.78**	− 0.84**	− 0.72**	0.16
	pH	0.04	0.26	− 0.28	0.09	0.09	0.06	0.00	− 0.14	− 0.07
	SOM on > 2 mm	0.02	− 0.02	− 0.01	0.06	− 0.02	− 0.25	− 0.11	− 0.26	− 0.26
	SOM on 2–0.25 mm	0.12	0.06	− 0.21	0.17	− 0.11	0.00	0.14	0.00	0.02
	SOM on < 0.25 mm	0.13	0.05	− 0.22	0.21	− 0.12	− 0.13	0.09	− 0.14	− 0.11

*Significant at α = 0.05; **Significant at α = 0.01.

## Discussions

### Influence of soil organic matter on zeta potential

In this study, the zeta potential of a clayey red soil was compared among 4 types of long-term treatments including manure, NPK + straw, NPK and CK in a subtropical climate. Generally, the manure treatment which also had the greatest concentration of SOC resulted in the highest clay zeta potential (less intense charge imbalance), while NPK + straw did not result in the second highest zeta potential as expected compared to the NPK and CK treatments. Variation in clay zeta potential among types of fertilization might be related with their different SOM content, because SOM had an influence on the zeta potentials via affecting the negative charges of soils^19^. The zeta potential of manure and NPK + straw treatments having high SOC agreed with earlier studies in Marchuk et al.^9^ that decreases of SOC via NaOH treatments decreased the negative zeta potential value^9^, where Claremont soil originally having high SOC (2.2%) displayed a greater degree of decline in negative zeta potential (from − 29 to − 34.9 mV) than Urrbrae having lower SOC (1.4%) (− 66.3 to − 68 mV). However, zeta potential in water dispersible clay responded to SOC contrastly in the study of Melo et al.^12^ , where Londrina soil with high SOC (5–20 g kg^−1^) displayed lower negative zeta potential values in water dispersible clay than that in Rondon soil (SOC 5 to 12 g kg^−1^) in subtropical Brazil.

Differences of SOC effect on zeta potential in our study and other studies were probably because ionic strength in bulk solution also affected the intensity of soil charge imbalance. Generally, in tropical and subtropical Ferralsols, high amounts of SOM that was released following the breakdown of macroaggregate provided an excess of negative charges and intensified the imbalance in charge, resulting in more negative in zeta potential of clay^12^. In contrast to Ferralsols in Brazil, red soil (highly-weathered) in our study showed higher negative zeta potential in manure soils with higher SOM. This was because high ionic strength in bulk solution might counterbalance the negative charges from SOM, and attenuated the imbalance in charges. Hence, manure treatment which provided greater EC and Ca^2+^, Mg^2+^ concentration and possibly higher ionic strength was reasonable to allow for more charge balance and greater negative zeta potential values than other treatment.

In this study, NPK + straw treatment exhibited similar negative zeta potential values as that in NPK but slightly lower than manure, probably due to the effect of SOM functional group from straw and soil solution concentration. Straw can increase the humin content as reported in the study of Sheng et al.^11^, and then a decrease of negative zeta potential can be induced as addition of humic acid on a Luvisol^20^. But the negative humic effect from straw on zeta potential was probably stronger than the positive effect from the increased bulk soil solution concentration in NPK + straw relative to NPK in Fig. 3 where increase of bulk solution concentration was found to increase the negative charge numbers and the negative zeta potential in Ultisol and Oxisol^15^. Therefore, our hypothesis that organic treatments decreased negative zeta potential value of soil was not supported for manure treatment, but was for NPK + straw treatment.

NPK + straw’s similar effect on negative zeta potential as NPK treatment was probably also related with their similar pH values. The effect of pH on the potential of clay surfaces can be related to the amount of variable charge on the external surface of the clay particles. Negative zeta potential decreased with rising pH of the solution due to deprotonation of the functional groups on the surface of the organic matter and Fe/Aloxides in NPK + straw treated soils. An increase of soil pH (from 3.5 to 7.5) influenced zeta potential through production of more negative net surface charges on soils in subtropical Australia^21,22^. Therefore, the pH in our study after KCl adjustment that showed a first increase and then decrease pattern with the increase of concentration, can help to explain the bell shape pattern of negative zeta potential (first decrease and then increase). However, in our study, the pH pattern with increment of KCl concentration was different from the results in study of Yu et al.^8^ where a continuous decline pattern in pH of two soils (Vertisol and Ultisol) was reported when the KCl concentration increased from 10^–5^ to 10^–1^ mol L^−1^. This is probably because the Ultisol possessed high amount of variable charges from Fe or Al oxides, which resulted in the diffusion layer attracted more positive charged cations (i.e. K^+^) from bulk solution to balance the increased negative charge on the surface of colloidal particles in order to maintain the electrical neutrality of the system^15^. This indicated that when KCl concentration was low, between 0 and 10^–2^ mol L^−1^, part of K^+^ was attracted to the diffuse double layer and the remaining K^+^ hydration allowed for raising in soil pH. When KCl concentration was beyond 10^–2^ mol L^−1^, many Al^3+^ions on soil exchange site were released into solution (0.03 to 0.12 mg L^−1^) through K^+^ exchange and probably dropped soil pH (data not shown).

Studies also found that the effect of SOM on zeta potential of clay also varied for soils in different climate. Yu et al.^8^ compared rice straw incorporation effect on two soils (Ultisol and Vertisol) and found that similar SOC content resulted in contrasting effects on surface potential of two types of soils, where surface potential of Ultisol continuously increased while firstly increased and became stable for Vertisol with increase of treated solution concentration. Different SOM effect on soil potential properties of two soils were probably associated with presence of soil variable charges in Ultisol^23^. SOM and Fe/Al (hydro)oxides in Ultisol carried a larger number of variable surface charges, and resulted in a strong overlapping of oppositely charged electric double layers (EDLs) between SOM and Fe/Al (hydro)oxides at low concentration^8^. The overlapping of oppositely charged EDLs between SOM and Fe/Al probably yielded in an increase in negative surface charge for Ultisols compared to Vertisol.

### Effect of SOM and zeta potential on soil aggregation

Increment in content of SOM after additions of straw or other organic treatments can improve aggregate stability^6,24,25^. The hydrophobic organic compounds that coated around soil particle can act as nucleus of aggregate formation and reduce the destruction effect from water infiltration^26,27^. The hydrophobic-C/hydrophilic-C increased from 1.04 to 1.07, from 1.22 to 1.27 for chicken manure and maize residues treatments, respectively, when soil water conditions changed from water deficiency to natural rainfall treatment^28^. This indicated that a small change of hydrophobic-C/hydrophilic-C might result in substantial change in soil water, which was a critical factor of aggregate development^28^. Xue et al.^24^ also reported that a small difference of aromatic percentage between tillage + straw and no tillage + straw treatments resulted in significant differences for aggregate (> 0.25 mm). Hence, small variation in soil hydrophobic-C groups can yield in soil aggregate variation. In our study, the manure treatment, which had higher SOM and hydrophobic-C (aromatic C) while lower hydrophilic-C than other treatments, was probably reasonable to yield in its higher stability than others. In these previous studies, the positive effect of SOM on soil aggregate development was attributed to the increment in van der Waals force between soil particles. However, different from our study, Melo et al.^12^ reported that Londrina soil with high SOC released greater water dispersible clay (60–80%) than that in Rondon with low SOC (50–70%) after mechanical breakdown of macroaggregate. This was probably due to the repulsive force prevailing attractive force between soil particles as affected by more negative zeta potential or surface potential^8^.

Clay zeta potential influenced the powerful electrostatic fields, soil internal forces and aggregate stability^9^. Decrease in negative clay zeta potential mainly yielded an increase in the soil microaggregate portion (< 0.25 mm, especially < 2 μm) when KCl concentration increased from 0 to 0.01 mol L^−1^ over all types of fertilization in our study, which was also confirmed by a negative correlation between zeta potential and grain percentage in Table 3. Similarly, decrease in surface potential of clay with increase in bulk solution (10^–4^ to 10^–2^ mol L^−1^) also increased the aggregate breaking strength for particles < 20 μm of a Loessal soil^29^. Disaggregation or clay dispersion with zeta potential was again probably attributed to the change in soil internal force. In these studies, when negative surface potential decreased, soil electrostatic repulsive force can exceed the Van der Waals attractive force, resulting in clay dispersion or aggregate blasting^29–31^. The soil disaggregation in water by slaking effect in the past studies was also thought to be actually originated from soil internal repulsive force which can reach about 100 MPa^32,33^.

### Implications and limitations

According to the aforementioned information, we claim that long-term manure application is a successful soil conservation practice in improving water stable macroaggregate stability (> 2 mm) of clayey red soils through increase in SOM content, aromatic functional groups, cations, and negative clay zeta potential. NPK + straw application was also beneficial for the increment in macroaggregate (> 2 mm), but has risk of releasing small soil particles (< 5 μm) due to decrease of negative clay zeta potential. NPK treatment did not show positive effect on red soil conservation. This indicated that the effect of fertilization on soils has limitations and the local climate should be taken into consideration before selection of type of fertilizer. Frequent wetting/drying in subtropical climate resulted in variations field soil concentration after fertilization application, as indicated by EC of 0 to 0.6 dS m^−1^ in our field. In precipitation, ions in the coarse soil pores that were wetted first by rain water can be transported to deeper soils^34^. Hence, the zeta potential probably can become more negative at the early stage of rainfall due to decrease in surface soil concentration in the field, affected soil particle dispersion and increased the risk of soil water erosion. To be noticed that, the zeta potential observed in the mechanically dispersed clay in the laboratory is not necessarily the same as spontaneous water dispersible clay in field conditions. But the results in the laboratory study can still help to elucidate the influence of zeta potential on soil aggregate stability after fertilization management to sustain ecological benefits for both farmers and policy-makers.

## Conclusions

Negative zeta potential of soil in response to KCl concentration depended on types of fertilization, where higher negative zeta potential value and its degree of variation occurred in manure than other treatments. NPK + straw had similarly low negative zeta potential value as NPK even though having higher SOM and aromatic-C functional groups than NPK. High SOM content and cation concentrations in manure treatment resulted in a greater soil pH and was probably responsible for more pH-dependent surface charge. Higher EC and amount of Ca^2+^ and Mg^2+^ cations from manure treatment together with SOM contributed to the higher zeta potential in manure relative to other treatments. As a result, manure helped to restrict the release of microaggregate (especially particles < 100 μm) but increased the portion of water stable macraggregates (> 2 mm) compared to NPK + straw, NPK and CK. The results indicated that long-term manure treatment can maintain clayey red soil aggregation through its greater extent of increase in negative zeta potential during a sharp decline in soil concentration in rain season.

## Materials and methods

### Study site description and soil samples collection

This study was conducted at an experimental station (30°01′N, 114°21′E) affiliated with the Huazhong Agricultural University in southeast Hubei, China. The local area had an annual mean temperature of about 16.8 °C and annual mean precipitation of about 1300 mm with most of the precipitation occurring from April to July. Different types of fertilizer treatments were applied annually into the research plots (each plot is 7 m × 3 m) for 17 years starting from 1998. The research design was a completely randomized block design with four treatments and three replicates, for a total of 12 plots. The different fertilizations included (1) chicken manure (manure) (10,000 kg ha^−1^ year^−1^), (2) NPK + rice straw (NPK + straw) (NPK + 1666 kg ha^−1^ year^−1^ rice straw), (3) inorganic fertilizers (NPK) (N: 175 kg ha^−1^ year^−1^ as CO(NH_2_)_2_; P: 150 kg ha^−1^ year^−1^ as Ca(H_2_PO_4_)_2_·2H_2_O; K: 115 kg ha^−1^ year^−1^ as KCl), and (4) control (CK, without any fertilizers). The NPK application rate was the same in (2) and (3) and was similar to local application rate, but the other two treatments application rates were higher than the “common criteria” (5000 kg ha^−1^ year^−1^) to satisfy the yields of corn (*Zea mays* L.) in the study site^35^.

At the study site, the red soils are classified as Ultisols using the USDA Soil Taxonomy system. In 2017, soil samples were collected from at least three random points from a depth of 0–20 cm under each type of fertilization in each plot. The samples were air-dried, crushed along the natural cracks, and dry-sieved through 8 mm sieves. The soils < 8 mm were deemed as bulk soil in this study and used for later KCl adjustment and subsequent water stable aggregate, pH and zeta potential analysis. General soil properties prior to collection of aggregate are reported in Table 4.

**Table 4:** The basic soil properties under different long-term fertilization treatments at 0–20 cm.

Treatments	Sand (%)	Silt (%)	Clay (%)	Bulk density (g cm^−3^)	pH	EC (dS m^−1^)	Saturated water content (cm^3^ cm^−3^)	Field capacity (cm^3^ cm^−3^)	Wilting point (cm^3^ cm^−3^)t	Alkaline hydrolisis N (mg kg^−1^)	Available P (mg kg^−1^)	Available K (mg kg^−1^)
Manure	15.3d	62.0a	22.7b	1.32b	6.4b	0.10a	0.50a	0.39a	0.27ab	87.2a	58.4a	156a
NPK + straw	27.6a	51.7b	20.7b	1.37b	6.7a	0.07b	0.48a	0.39a	0.29a	58.1b	31.0b	138b
NPK	19.6c	47.4c	33.0a	1.46a	6.6a	0.07b	0.45b	0.35ab	0.25b	57.1b	34.3b	109c
CK	23.3b	53.9b	22.8b	1.35b	6.2c	0.08b	0.49a	0.33b	0.23b	60.6b	16.7c	80.8d

Different lowercase letters indicate the same soil parameter in column was significantly different within treatments.

### Soil organic matter analysis

The soil organic carbon (SOC) and soil organic matter (SOM) on each aggregate size (> 2, 2–0.25 and < 0.25 mm) were determined by the K_2_Cr_2_O_7_–FeSO_4_ oxidation method. The three aggregate size portions here were wet-sieved from aggregates size (5 to 2 mm) in bulk soil. The humic acid of bulk soil under each fertilization was obtained following the procedure in International Humic Substances Society (IHSS), and then the major C functional groups of humic acid were analyzed by CP/MAS ^13^C-NMR. The C functional groups was realized according to the chemical shift, with 0–45 ppm chemical shift as Alkyl-C, 45–60 ppm as Methoxyl /N-alkyl-C, 60–110 ppm as O-alkyl-C, 110–160 ppm as Aromatic-C, and 160–220 ppm as Carbonyl-C^27^. The areas of the absorption bands in the CP/MAS ^13^C-NMR spectra were integrated using the OPUS 5.5 software and were defined as intensities.

### Bulk soil KCl adjustment

Bulk soil samples (< 8 mm) were evenly packed into PVC column (diameter = 10 cm; height = 5 cm) to reach a bulk density of 1.3 g cm^−3^, with the column base being fitted with nylon mesh screens. Soils in columns were treated by different KCl concentration (0, 0.001, 0.01, 0.05, and 0.1 mol L^−1^) to adjust to various EC and pH following below procedures. All solutions were prepared using distilled water. The soil columns were gradually wetted by KCl solution in an upward direction for 24 h, followed by being drained for 24 h. The wetting and draining cycles of soil treatment was repeated for 3 times for each of solutions. After that, the soils were allowed to equilibrate for 1 month in a climate chamber (at constant temperature of 25 °C, constant moisture of 60%). Finally, the soils were removed from the PVC columns, air-dried, crushed, and passed through a sieve to obtain 5–2 mm aggregates. These 5–2 mm aggregates after KCl treatment were separated into three parts of samples, part (1) sample for further analysis of water stable aggregate size distribution, part (2) sample for zeta potential, and part (3) sample for pH and cations.

### Soil aggregate size distribution by sieving and laser diffraction analysis

After KCl adjustment, the soil aggregate (5–2 mm) in part (1) were then analyzed for water-stable aggregate size distribution and aggregate stability. 10 g of soils (5–2 mm) were laid on the top of a series of sieves assembled as 2 and 0.25 mm, and then submerged into the distilled water to gently shaking for 20 times with 2 cm in amplitude. After that, the soil aggregates retained on each sieve were dried for at least 48 h at 40 °C and weighed to determine the aggregate size distribution. Each soil aggregate determination was repeated for five times. Aggregate mean weight diameter (MWD) was also calculated as Eq. (1).

1 $$MWD = \sum\limits_{1}^{n + 1} \frac{r_{i - 1}+r_{i}}{2} + m_{i}$$

where, in Eq. (1), *r* = aperture of the *i*th mesh (mm), *r*_*0*_ = *r*_*1*_, and *r*_*n*_ = *r*_*n*+*1*_; *m*_*i*_ = mass fraction of aggregates remaining on *i*th sieve; *n* = number of the sieves.

To further illustrate the small soil particle distribution after each treatment, only soils (< 2 mm) was re-analyzed using the Mastersizer3000 equipment. Soils were treated following below procedure. Soils were shaken in water and centrifuged, followed by supernatant decanting. The procedure was repeated until the EC of the supernatant reached the value of distilled water. After that, the soil solution pH was adjusted to 7 and shaken for 2 h to use laser diffraction technique in wet mode for analysis (water dispersion; the particle absorption index was 0.02, particle refractive index was 1.60 and dispersant refractive index was 1.33). A background measurement was performed firstly to subtract the ambient light signal from the total scattered light received from the sample, followed by three consecutive analyses of soil sample per lens. Finally, the results display the volume content (%) distribution of particles ranging from 0.01 to 3000 μm in 100 bin. The data of three measurements were averaged to obtain relative volume data.

### Soil clay zeta potential, pH and cations

After KCl adjustment, 5 g of soil (5–2 mm) in part (2) was shaken with 100 cm^3^ of distilled water for 2 h. After shaking, the suspensions were transferred to a 1 L graduated cylinders, volume brought to 1 L, vertically mixed, and particles allowed to settle. Clay particles (< 2 μm) were pipetted (10 mL) from 15 cm below the water surface after having settled for 11 h and 37 min according to the Stoke’s Law. The zeta potential of clay particles (< 2 μm) was analyzed using Malvern Zeta master Particle Electrophoresis Analyzer. Another set of soils from part (3) were ground, sieved through 2 mm, and analyzed for pH_1:1_ at 1:1 soil to water solution. The Ca^2+^ and Mg^2+^ concentration in soils from part (3) were extracted by ammonium acetate solution and determined by ICP-OES. Each chemical analysis was repeated three times.

### Statistical analysis

The model’s residuals’ normal distribution was tested. Differences of clay zeta potential, SOM, and aggregates size percentage among each solution concentration under each type of fertilization were assessed using Fisher's least significant difference (LSD) test (α = 0.05). Pearson correlation among all the soil parameters was also determined in SPSS 17. All the Figures were plotted by Origin 8.0.
